# Near-infrared luminescent probes for bioimaging and biosensing

**DOI:** 10.1039/d1sc90046c

**Published:** 2021-03-17

**Authors:** Fan Zhang, Ben Zhong Tang

**Affiliations:** Department of Chemistry, Shanghai Key Laboratory of Molecular Catalysis and Innovative Materials, State Key Laboratory of Molecular Engineering of Polymers, iChem, Fudan University Shanghai 200433 P. R. China zhang_fan@fudan.edu.cn; Department of Chemistry, The Hong Kong Branch of Chinese National Engineering Research Center for Tissue Restoration and Reconstruction, Institute for Advanced Study, Department of Chemical and Biological Engineering, The Hong Kong University of Science and Technology Clear Water Bay Kowloon Hong Kong China tangbenz@ust.hk; Center for Aggregation-Induced Emission, SCUT-HKUST Joint Research Institute, State Key Laboratory of Luminescent Materials and Devices, South China University of Technology Guangzhou 510640 China; AIE institute Guangzhou Development District, Huangpu Guangzhou 510530 China

## Abstract

Fan Zhang and Ben Zhong Tang introduce the *Chemical Science* themed issue on Near-infrared luminescent probes for bioimaging and biosensing.
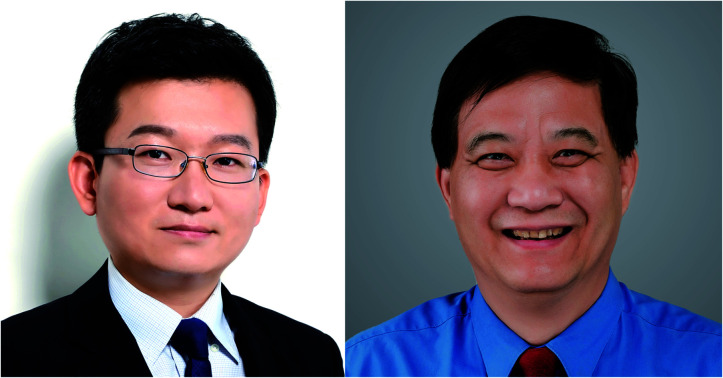

Near-infrared (NIR) fluorescence imaging (wavelength range: 650–1700 nm) is one of the remarkable imaging modalities for improved bioimaging and biosensing in both fundamental research and clinical applications, owing to its high spatiotemporal resolution and non-invasiveness. It has been a growing research topic for decades and will continue to flourish in the coming years. In this themed issue, selected studies focused on NIR bioimaging and biosensing published in *Chemical Science* in the past 18 months have been collected. Through this themed issue, we aim to call on the research community to strengthen cooperation with scientists among diverse research fields, such as chemistry, biology and biomedicine.

First of all, the imaging agent is the cornerstone of fluorescence bioimaging. Up to now, different kinds of fluorescence bioimaging agents have flourished, including fluorescent dyes (DOI: 10.1039/C8SC00900G), polymers (Pdots) (DOI: 10.1039/C8SC03510E), covalent organic nanosheets (DOI: 10.1039/C8SC02842G), rare-earth nanoparticles (DOI: 10.1039/C8SC05044A, DOI: 10.1039/C8SC00927A), metal complexes (DOI: 10.1039/C8SC00259B), *etc.* With diversified imaging materials, different applications may be achieved. For example, nanoparticles are good imaging agents for multicolour bioimaging and bio-coding with excellent photostability. At the same time, organic fluorescent dyes are bright and structurally compact with lower toxicity concerns. Ongoing research is focused on the development of new fluorescent dyes with outstanding properties (DOI: 10.1039/C9SC02314C, DOI: 10.1039/C8SC00089A) and finds more practical applications with various imaging agents.

For fluorescence bioimaging, the longer wavelength can penetrate more deeply into bio-tissues owing to the reduced scattering and autofluorescence. To achieve this goal, researchers try to red-shift the imaging wavelength to the NIR-II window (1000–1700 nm). For example, Hong and co-workers reported a small molecule NIR-II fluorescent dye, realizing deep imaging of the gastrointestinal tract in both healthy and diseased mice models (DOI: 10.1039/C8SC04363A). Cheng and co-workers developed Pdots as NIR-II fluorescent probes for image-guided orthotopic tumor surgery (DOI: 10.1039/C8SC00206A). In addition to organic molecules, NIR-II probes based on inorganic materials have also been explored. For example, Chen reported rare-earth nanoparticles (NaCeF_4_:Er/Yb nanocrystals), which exhibited high sensitivity for uric acid detection and excellent resolution for *in vivo* imaging (DOI: 10.1039/C8SC00927A). It is worth noting that although NIR-II probe systems have made huge achievements possible recently, the performances of these systems, such as the fluorescence quantum yield and wavelength, are yet to be further improved. On the other hand, two-photon excitation is an alternative method to get images in deep tissues (DOI: 10.1039/C8SC04685A).

In addition, better imaging performance can also be obtained by novel imaging modalities such as chemiluminescence, lifetime, afterglow, room-temperature phosphorescence (RTP) imaging, *etc.*, because of their almost zero-autofluorescence background. For instance, Laursen and Zhang independently reported time-gated imaging in cells using long-lived fluorophores (DOI: 10.1039/C8SC00259B, DOI: 10.1039/C8SC00089A). Ma and coworkers developed NIR-emissive RTP materials, which provided a useful platform for NIR imaging (DOI: 10.1039/C9SC05502A). Zhang and co-workers reported an afterglow system with enhanced afterglow intensity and prolonged the afterglow duration for tumor imaging (DOI: 10.1039/C9SC04901K). Fan and Shabat independently developed supramolecular complexes as chemiluminescence probes for targeting bioimaging (DOI: 10.1039/C8SC04012E, DOI: 10.1039/C8SC05174G). These novel imaging modalities may find further use in various biological applications.

Fluorescence bioimaging allows us to picture targets with ultrahigh resolution. At the same time, it is also urgent to realize biosensing, which requires the fluorescent probe to be able to respond to the specific stimuli selectively. Generally speaking, probes that operate in luminescence turn-on and ratiometric modes are widely used and are more reliable for accurate analyte sensing. In the former case, Tan and coworkers developed an aptamer structure change-induced fluorescence turn-on probe based on metal–organic framework (MOF) nanoparticles (DOI: 10.1039/C8SC02210K). Zhao and Fan independently reported enzyme-activated turn-on probes (DOI: 10.1039/C8SC04685A, DOI: 10.1039/C9SC02093D). Pu and coworkers reported a ROS/RNS probe that could specifically distinguish keloid fibroblasts (DOI: 10.1039/C8SC01865K). On the other hand, Zhang and Ahn developed ratiometric fluorophores for cell imaging (DOI: 10.1039/C8SC01673A, DOI: 10.1039/C9SC02287B). However, biosensing in deep tissue is still in its infancy. Future works focused on quantitative biosensing in deep tissue are encouraged.

Many fluorophores may suffer from aggregation-caused quenching (ACQ) in aqueous solution, which limits the imaging quality in biological applications. In contrast, fluorophores with the aggregation-induced emission (AIE) phenomenon emit intensely when aggregated in aqueous solution because of the restriction of the intramolecular motion (RIM) mechanism (DOI: 10.1039/C7SC04820C). Therefore, AIE fluorophores with high brightness may find broad applications in bioimaging. For example, researchers have shown that AIE dots can be used for non-invasive and real-time NIR-II imaging of the gastrointestinal tract in health and disease situations (DOI: 10.1039/C8SC04363A). Chan and co-workers reported NIR Pdots based on the molecular design strategy of AIE for specific tumor targeting (DOI: 10.1039/C8SC03510E). Since the coinage of the concept, AIE has gradually changed people’s way of thinking about luminogen aggregation and brought about a revolution of luminogen research both conceptually and technically.

Last but not least, NIR probes can also be used for image-guided therapy (DOI: 10.1039/C9SC02466B), such as photo-controlled drug release (DOI: 10.1039/C8SC04012E, DOI: 10.1039/C7SC05414A), photothermal therapy (PTT) and photodynamic therapy (PDT) (DOI: 10.1039/C8SC02210K, DOI: 10.1039/C9SC04034J). Image-guided surgery is also a promising application of fluorescence bioimaging. With bright and stable fluorophores, NIR-II fluorescence-guided surgery may become an ideal modality for clinical usage since NIR-II fluorescence is barely affected by operation room light (DOI: 10.1039/C8SC00206A).

This themed issue is a very concentrated selection of interesting studies of fluorescence bioimaging and biosensing showing the development of new fluorescent dyes, NIR fluorescence bioimaging, two-photon excitation fluorescence bioimaging, NIR-II bioimaging and biosensing, image-guided therapy, image-guided surgery and so forth. It is a growing field, which will boost the integration of chemistry and biomedicine and finally benefit the clinic.

We hope this themed issue sparks further inspiration for the development of new NIR theranostic systems and explanations of their applications. We believe that it will not only draw new researchers into this promising field but also inspire veteran researchers to drive NIR probes to a higher stage. It has been a pleasure to put together this themed issue and we hope that the readers of *Chemical Science* enjoy reading it as much as we have enjoyed compiling it.

Professor Fan Zhang and Professor Ben Zhong Tang

*Guest Editors*

